# Incidence and outcomes of spinopelvic fixation loosening following adult spinal deformity surgery

**DOI:** 10.1007/s43390-026-01281-6

**Published:** 2026-06-16

**Authors:** John D. Arena, Yohannes Ghenbot, Mert Marcel Dagli, Joshua L. Golubovsky, Ben Gu, Addison Quinones, Dominick Macaluso, Neil R. Malhotra, Zarina S. Ali, Brendan F. Judy, Jang W. Yoon, William C. Welch, Vincent Arlet, John H. Shin, Ali K. Ozturk

**Affiliations:** 1 Department of Neurosurgery, Perelman School of Medicine, University of Pennsylvania, Philadelphia, PA USA; 2 Department of Orthopaedic Surgery, Perelman School of Medicine, University of Pennsylvania, Philadelphia, PA USA

**Keywords:** Spine deformity, Pelvic fixation, Iliac screw, S2AI screw, Kyphosis, Scoliosis

## Abstract

**Purpose:**

To identify the incidence and clinical outcomes of spinopelvic fixation loosening following surgical correction of adult spinal deformity (ASD).

**Methods:**

A single-institution retrospective cohort of 152 consecutive patients meeting inclusion criteria who underwent long-segment fusion with pelvic fixation for correction of ASD was reviewed. Pelvic screws were assessed on follow-up post-operative standing radiographs for evidence of loosening. Surgical revision of spinal instrumentation served as the primary outcome. PROMIS Pain Interference and Physical Function T-Scores were assessed as secondary outcomes among the subset of cases (*n* = 92) with available scores.

**Results:**

Pelvic screw loosening was appreciated in 25 cases (16.4%) with initial evidence of loosening identified a median 1.1 years (Q1: 0.8, Q3: 2.5) following surgery. Loosening of pelvic screws (OR 3.0, 95% CI 1.2–7.3, *p* = .017) was associated with significantly increased odds of eventual instrumentation revision surgery, including specifically surgical revision of spinopelvic fixation (OR 5.5, 95% CI 1.6–18.5, *p* = .006). 4 of 26 cases (15%) with S2-Alar-Iliac (S2AI) screws required revision surgery compared to 37 of 126 cases (29%) without S2AI screws. PROMIS T-Scores were significantly worse for patients requiring instrumentation revision surgery but did not differ between cases with and without loosening.

**Conclusion:**

Loosening of spinopelvic fixation occurs frequently following correction of ASD and can develop years following surgery. While distal construct screw loosening may be appreciated incidentally at follow-up, loosening of pelvic screws was associated with significantly increased odds of eventual instrumentation revision surgery.

## Introduction

Adult spinal deformity (ASD) is a common and debilitating condition, frequently associated with severe back pain and functional impairment [1]. ASD often develops in the setting of progressive degenerative changes or iatrogenic flat back [2]. As the population ages and rates of spinal fusion surgery increase, the prevalence of ASD is expected to continue to grow [3, 4]. Surgical correction of ASD may confer considerable improvement in quality of life, however, long-segment fusion has been associated with significant morbidity due to a high rate of post-operative complications [5].

Failure of spinal instrumentation occurs frequently following correction of ASD and often requires surgical revision. However, identifying patients at risk for complications remains challenging [6, 7]. Notably, high rates of distal segment screw loosening have been reported following ASD correction, where spinopelvic fixation with iliac and/or S2-alar-iliac (S2AI) screws is performed to stabilize long-segment fusion constructs [8–10]. In contrast to screw loosening in the context of acute distal failure, the clinical ramifications of incidentally identified screw loosening warrant consideration. Such loosening of spinopelvic fixation may be identified on routine post-operative standing radiographs as radiolucency around screws. The development of proximal screw loosening following ASD correction has been associated with significantly increased odds of eventual instrumentation failure requiring revision surgery [11].

Herein, we examine the impact of distal construct loosening within this population. Specifically, we assess the incidence and clinical outcomes of spinopelvic fixation loosening in a retrospective institutional cohort of patients who underwent long-segment fusion for the correction of ASD.

## Materials and methods

### Patient population

**Fig. 1:**
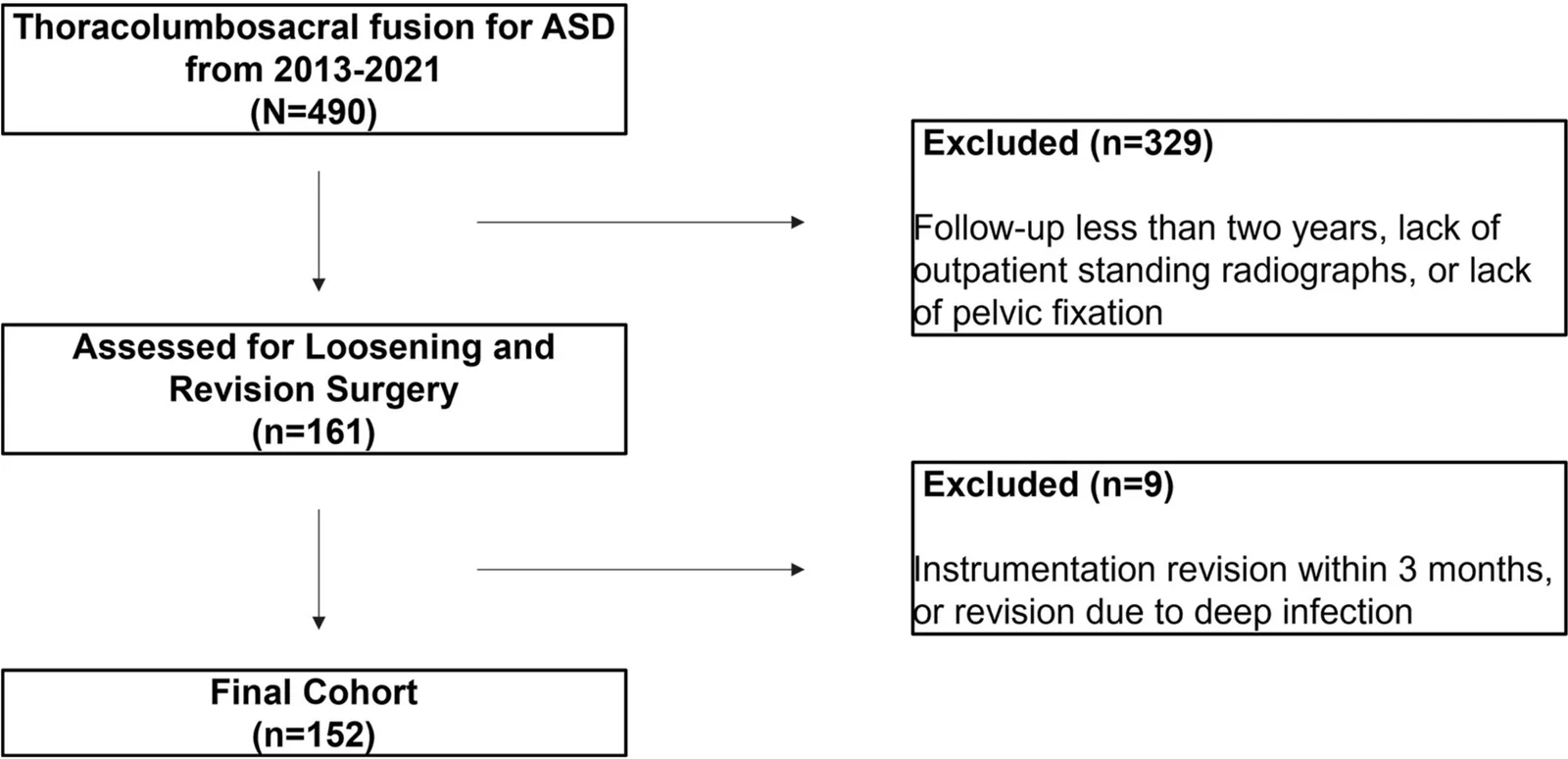
STROBE cohort inclusion flowchart

A single-institution retrospective analysis was performed on a cohort of adult patients who underwent long-segment fusion for the correction of ASD. The findings are presented in accordance with STROBE recommendations [12]. Of 490 consecutive cases who underwent index thoracolumbosacral fusion involving ≥ 6 vertebrae for the correction of deformity between 2013 and 2021, we included those with minimum two-year post-operative follow-up and who obtained routine outpatient follow-up standing radiographs. Cases without spinopelvic fixation to the ilium were excluded. Cases with early short-interval instrumentation failure occurring within three months of index surgery were excluded. Cases with delayed loosening found to have deep infection were excluded. All patients meeting the above inclusion criteria between 2013 and 2021 were considered and cases were only excluded based upon pre-defined criteria. Ultimately, 152 cases were included in the cohort for analysis (Fig. 1).

Approval for this study was provided by the Institutional Review Board of the University of Pennsylvania (Protocol number 848854). Patient age, sex, body mass index (BMI), smoking status and bone health (osteopenia or osteoporosis) at the time of surgery were identified through retrospective chart review. The cohort includes patients managed by multiple different surgeons within our institution. As such, pre-operative screening and optimization practices were variable. For patients with available pre-operative DEXA scans, bone density scores were utilized in the determination of osteoporosis/osteopenia status. For patients without pre-operative DEXA scans, records were reviewed for documented history of osteoporosis or osteopenia. Surgical details including levels of instrumentation, use of L5-S1 interbody cage, number of pelvic screws, type of pelvic screws (traditional iliac screws and/or S2AI screws), number or rods, and performance of three column osteotomy were determined through review of operative reports and post-operative radiographs.

### Outcome measures

**Fig. 2:**
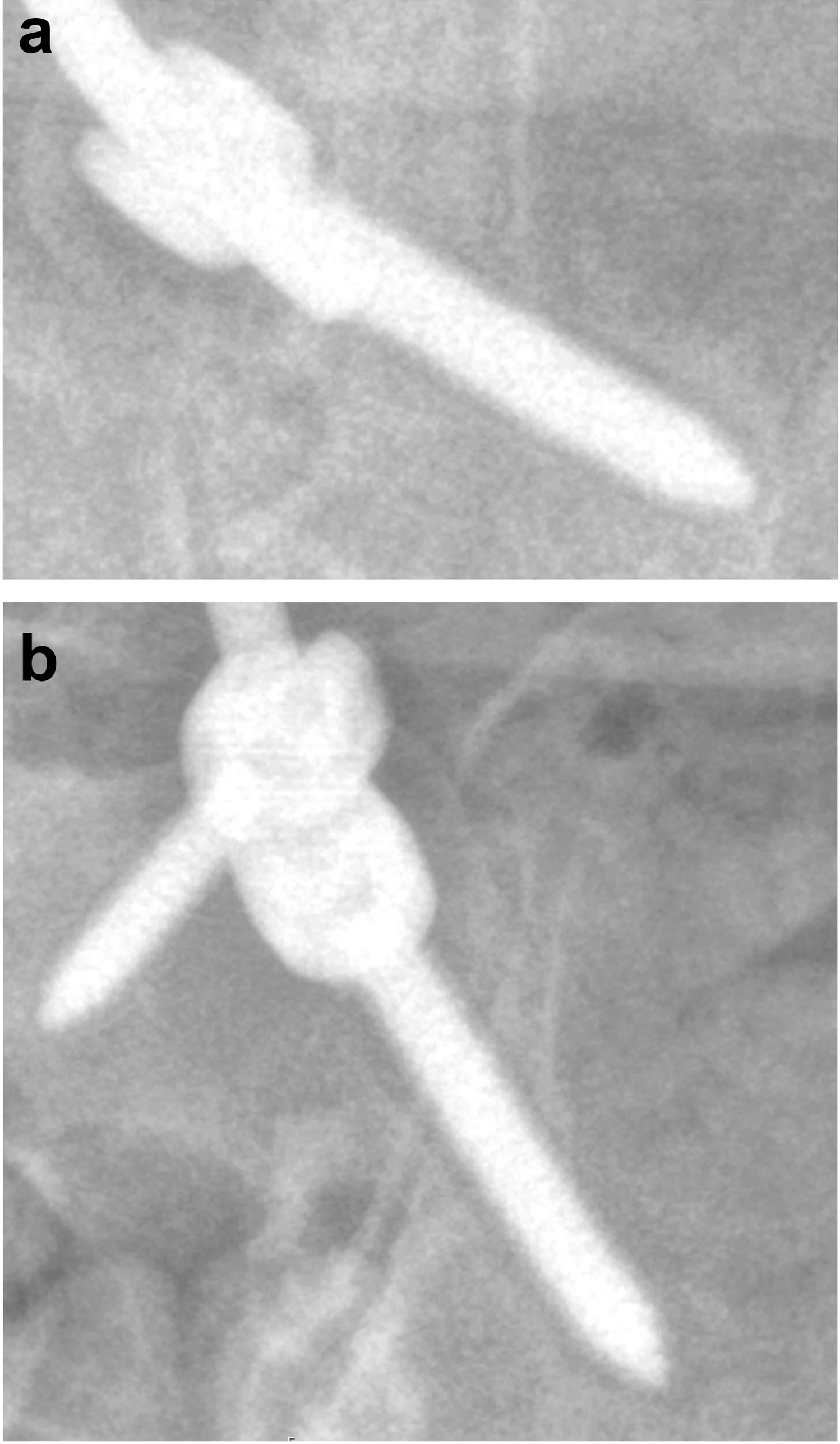
Loosening of spinopelvic fixation. **a**, **b** Evidence of pelvic screw loosening with radiolucency surrounding the screw threads on follow-up post-operative standing radiographs

All patients were assessed in post-operative follow-up with standing whole spine radiographs. All available post-operative radiographs were reviewed for each case, regardless of clinical outcome. Spinopelvic fixation instrumentation was assessed for evidence of loosening on anteroposterior (AP) and lateral views of post-operative follow-up standing radiographs. Specifically, pelvic screws were evaluated in both views for the presence of surrounding radiolucency (Fig. 2). Screw loosening was assessed by two spine surgery fellows, who served as independent reviewers (JDA and YG), blinded to clinical outcome, with very good inter-rater reliability (Cohen’s Kappa 0.77) [13, 14]. Post-operative CT scans were not routinely obtained in the follow-up management of patients in the cohort. In light of this, post-op CTs, if available for any given case, were not utilized in the assessment of screw loosening in order to allow standardized assessment of instrumentation integrity across the cohort.

Spinal instrumentation revision surgery served as the primary outcome. For patients who did not require instrumentation revision surgery, study endpoints were defined as the most recent available follow-up radiographs and patient reported outcome measures. For patients who did require revision surgery, study endpoints were defined as the most recent available data preceding the date of instrumentation revision. Spinal implant and surgical supply costs for instrumentation revision surgeries were extracted from the electronic medical record.

A subset of patients completed the Patient Reported Outcomes Measurement Information System (PROMIS) Pain Interference v1.1 and PROMIS Physical Function v2.0 measures [15] as part of their routine outpatient care. PROMIS T-Scores were available for 92 of 152 (60.5%) patients post-operatively. Among the subset of patients with available data, PROMIS Pain Interference and Physical Function T-scores were assessed as secondary outcomes.

### Statistical analyses

Continuous data are presented as means ± standard deviations (SD), or medians alongside 25th percentile (Q1) and 75th percentile (Q3) values. Categorical data are presented as number (*n*) and percentage of total. *T* Tests were used for comparisons of continuous data between groups. Multivariate logistic regression analyses were performed to assess the odds of binary outcomes, with adjustment for smoking status and bone health. Odds ratios are presented alongside 95% confidence intervals (CI) and associated *p* values. Kaplan–Meier failure estimate survival analyses were performed for screw loosening and instrumentation revision. Differences in failure curves were assessed via the log-rank test [16]. Two-sided *p* values < 0.05 were considered statistically significant. Statistical analyses were performed with STATA 15.1 Statistical Software (College Station, Texas).

## Results

One hundred and fifty-two patients underwent thoracolumbosacral fusion with pelvic fixation for correction of ASD. The cohort was comprised of 104 females (68.4%) and 48 males (31.6%) with a mean age of 64.1 years (SD ± 9.0) and mean BMI 27.6 (SD ± 5.2). 26 patients (17.1%) were active smokers at the time of index surgery. Osteoporosis was diagnosed pre-operatively in 28 cases (18.4%) and osteopenia in 45 cases (29.6%). In terms of spinal constructs, 57 cases (36.8%) ended proximally in the upper thoracic spine (T1-T6) and 95 (62.5%) ended in the lower thoracic spine (T7-12). Posterior supplementary rods were utilized in 43 cases (28.3%). Interbody fusions were performed at L5-S1 in 103 cases (67.8%). Of the 103 L5-S1 interbody fusions, 88 were performed via ALIF and 15 via TLIF. Among the cases with L5-S1 interbody fusion, 87 (84.5%) had bilateral pedicle screws at both L5 and S1. All cases demonstrated distal construct pelvic fixation with 1 (12 cases, 7.9%), 2 (107 cases, 70.4%), 3 (9 cases, 5.9%) or 4 (24 cases, 15.8%) pelvic screws. Iliac screws were utilized in 126 (82.9%) cases, S2-Alar-Iliac (S2AI) screws in 7 (4.6%) cases, and combination of iliac and S2AI in 19 (12.5%) cases. Patients were followed for a median 4.4 years (Q1: 2.9, Q3: 5.8).

### Incidence of pelvic screw loosening

**Fig. 3:**
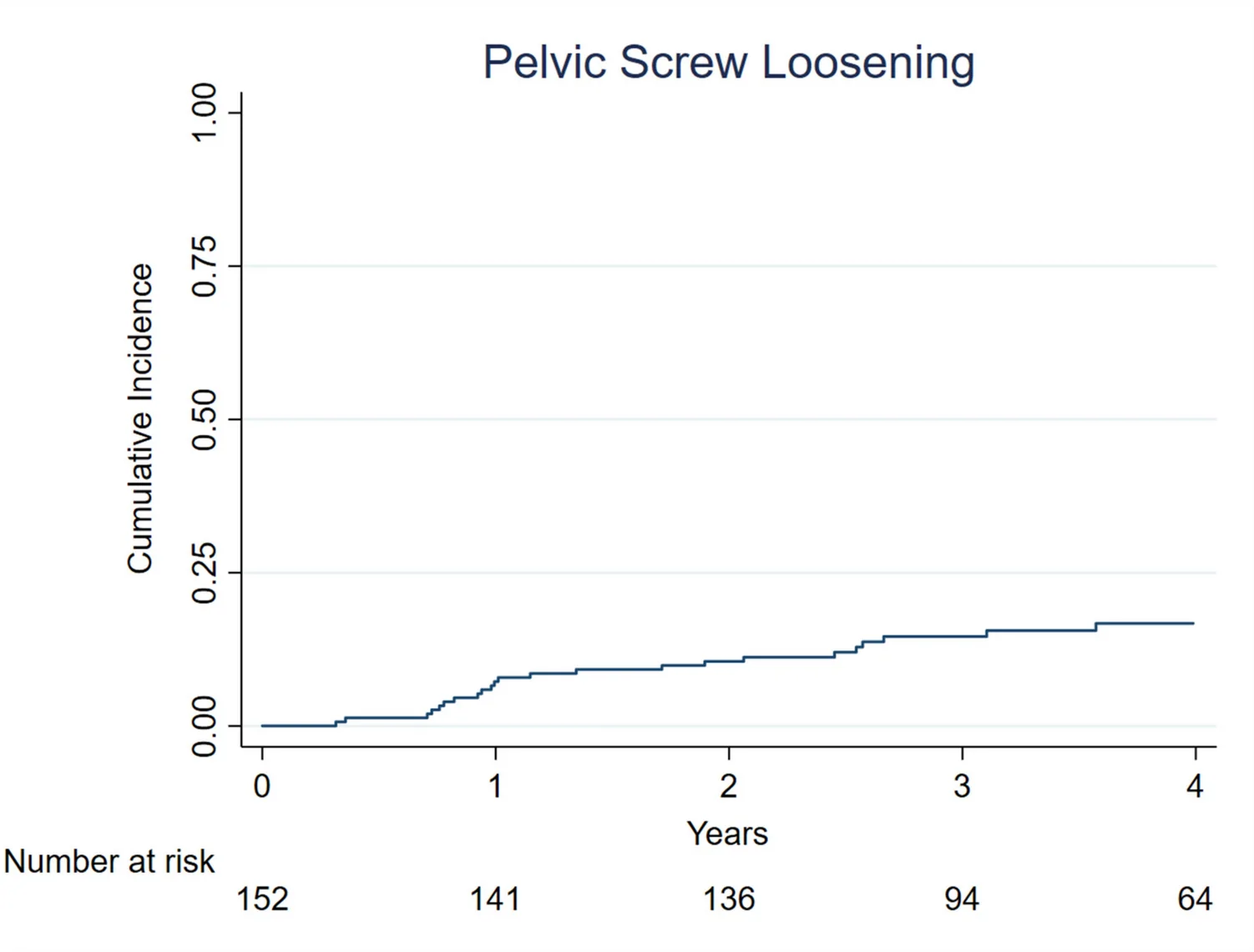
Cumulative incidence of pelvic screw loosening. Kaplan–Meier failure analysis curves demonstrate the cumulative incidence of pelvic screw loosening over time. The number of remaining cases at risk are listed below each timepoint to account for censoring of cases without further follow-up

The majority of cases (*n* = 127, 83.6%) did not demonstrate any evidence of pelvic screw loosening on outpatient follow-up radiographs. Pelvic screw loosening was appreciated in 25 cases (16.4%), with initial identification occurring a median 1.1 years (Q1: 0.8, Q3: 2.5) following index deformity correction surgery. Demographic and surgical features of patients with and without pelvic screw loosening are displayed in Table 1. Kaplan–Meier failure function analysis estimates a cumulative incidence of pelvic loosening of 0.07 (95% CI 0.04–0.13) at one year, 0.11 (95% CI 0.07–0.17) at two years and 0.15 (95% CI 0.10–0.22) at three years following surgery **(**Fig. 3**)**.

**Table 1:** Patient demographics and surgical features in pelvic screw loosening

	No pelvic screw loosening (*n* = 127)	Pelvic screw loosening (*n* = 25)
Age in years (Mean ± SD)	64.3 ± 8.8	62.8 ± 9.7
Sex		
Male	40 (31.5%)	8 (32.0%)
Female	87 (68.5%)	17 (68.0%)
BMI	27.4 ± 5.0	28.8 ± 6.0
Smoker	20 (15.7%)	6 (24.0%)
Bone quality		
Osteopenia	40 (31.5%)	5 (20.0%)
Osteoporosis	22 (17.3%)	6 (24.0%)
UIV		
Upper thoracic (T1-6)	47 (37.0%)	10 (40.0%)
Lower thoracic (T7-12)	80 (63.0%)	15 (60.0%)
Number of pelvic screws		
1	10 (7.9%)	2 (8.0%)
2	89 (70.1%)	18 (72.0%)
3	6 (4.7%)	3 (12.0%)
4	22 (17.3%)	2 (8.0%)
Pelvic screw construct		
Iliac screws	105 (82.7%)	21 (84.0%)
S2AI screws	5 (3.9%)	2 (8.0%)
Iliac and S2AI screws	17 (13.4%)	2 (8.0%)
Rods		
2	89 (70.1%)	20 (80.0%)
3	11 (8.7%)	3 (12.0%)
4	26 (20.5%)	2 (8.0%)
6	1 (0.8%)	0 (0.0%)
L5-S1 interbody cage	85 (66.9%)	18 (72.0%)
Three column osteotomy	5 (3.9%)	1 (4.0%)
Median follow up in years (Q1, Q3)	3.8 (2.6, 5.5)	5.2 (4.1, 6.3)

*BMI* body mass index; *Q1* first quartile (25th percentile); *Q3* third quartile (75th percentile); *S2AI* S2-Alar-Iliac; *SD* standard deviation; *UIV* Upper instrumented vertebra

4 of 26 (15.4%) cases with S2AI screws developed pelvic screw loosening compared to 21 of 126 (16.7%) cases with only traditional iliac screws. Pelvic screw loosening was identified in 15.2% of cases with supplementary (> 2) pelvic screws compared to 16.8% of cases with 1 or 2 pelvic screws. Pelvic screw loosening was appreciated in 17% of cases with L5-S1 interbody cage compared to 14% of cases without L5-S1 interbody fusion. Among the subset with ALIF at L5-S1 (*n* = 88), loosening of pelvic screws was appreciated in 16 cases (18%). 9 of 64 cases (14%) without ALIF developed loosening of spinopelvic fixation. Pelvic screw loosening developed in 6 of 28 (21%) of patients with osteoporosis and 5 of 45 (11%) with osteopenia compared to 14 of 79 (18%) with normal bone density.

### Instrumentation revision surgery following deformity correction

Following long-segment fusion for correction of ASD, forty-one patients (27.0%) eventually required instrumentation revision surgery. Revision surgeries were performed a median 2.7 years (Q1: 1.1, Q3: 3.6) following index deformity correction. Complete list of indications for instrumentation revision are listed in Table 2. Spinal implant and surgical supplies, alone, cost an average of $15,467 (SD ± $11,845) per revision surgery.

**Table 2:** Indications for instrumentation revision surgery

Rod fracture with pseudoarthrosis	17
Painful instrumentation	12
Proximal junctional kyphosis	8
Rod dislodged	1
Pseudoarthrosis without rod fracture	1
Additional deformity correction	1
Adjacent segment disease	1
Total	41

Cases requiring revision surgery were younger at the time of index surgery (61.5 ± 9.4 years vs. 65.1 ± 8.6 years) and had slightly higher BMI (28.3 ± 5.2 kg/m^2^ vs. 27.4 ± 5.2 kg/m^2^). A greater percentage of patients requiring revision surgery were active smokers (24.4% vs 14.4%). In considering surgical factors, 4 of 26 cases (15%) with S2-Alar-Iliac (S2AI) screws required revision surgery compared to 37 of 126 cases (29%) without S2AI screws. Among cases with L5-S1 ALIF, 26 of 88 cases (29.5%) ultimately required instrumentation surgery, of which 9 involved revision of spinopelvic fixation. Instrumentation revision was performed in 15 of 64 cases (23.4%) without ALIF, including 4 instances of revision of spinopelvic fixation. Patients requiring revision were also followed for a longer duration after index surgery (mean 5.3 ± 1.7 vs 4.0 ± 1.6 years). Among the subset of patients with available PROMIS T-Scores, those requiring instrumentation revision surgery demonstrated significantly worse pain interference (mean difference 5.3, 95% CI 2.0 to 8.7) and physical functioning (mean difference − 3.6, 95% CI − 7.0 to − 0.3).

### Outcomes following loosening of spinopelvic fixation

Among the 25 patients with evidence of pelvic screw loosening, 12 (48%) ultimately required instrumentation revision surgery, including 6 instances of revision of spinopelvic fixation. Pelvic screw loosening was associated with significantly increased odds of instrumentation revision surgery (OR 3.0, 95% CI 1.2–7.3, *p* = 0.017), including specifically surgical revision of spinopelvic fixation (OR 5.5, 95% CI 1.6–18.5, *p* = 0.006). Revision surgeries were performed a median 73 days (Q1: 33, Q3: 277) following initial radiographic evidence of loosening.

**Fig. 4:**
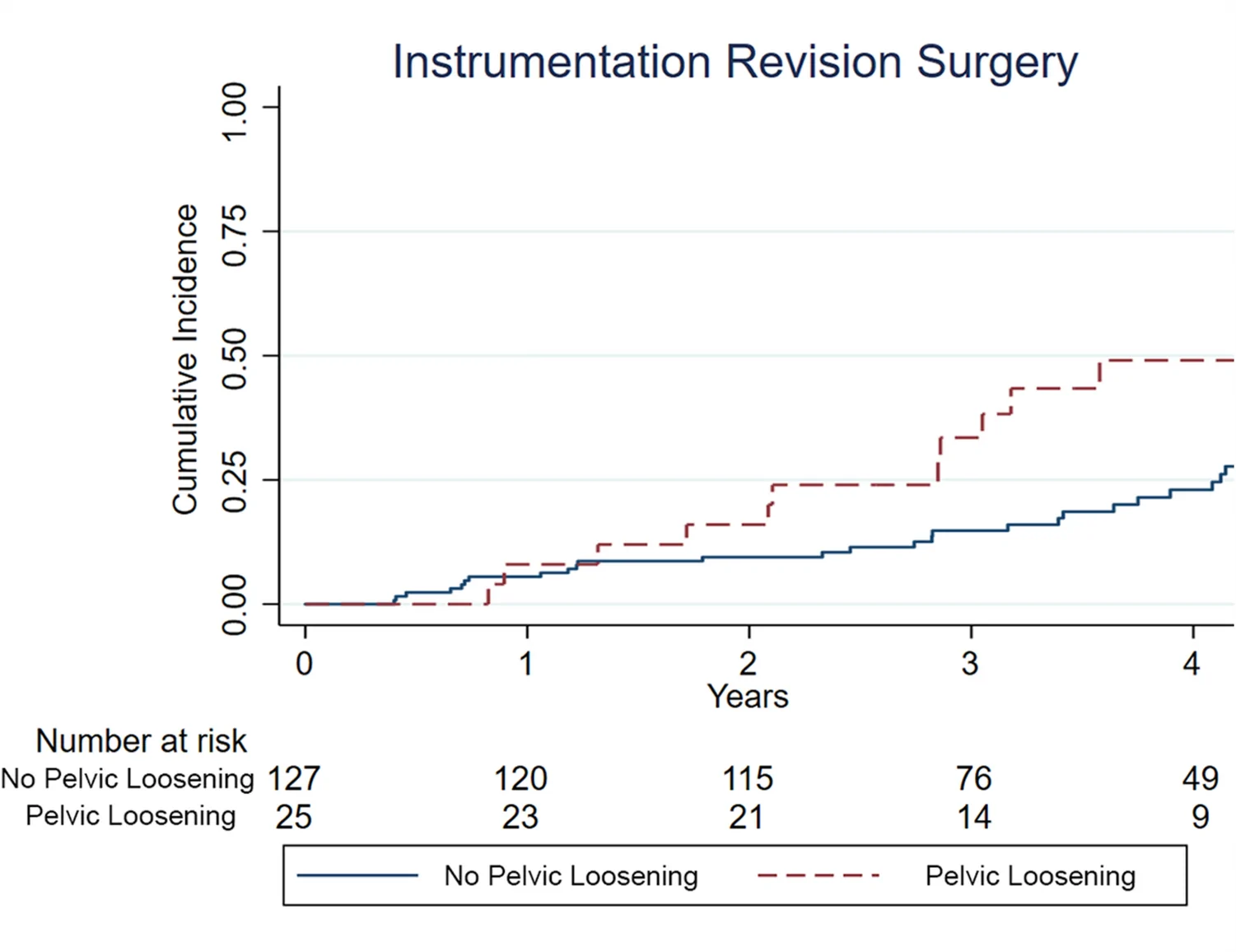
Cumulative incidence of instrumentation revision following pelvic loosening. Kaplan–Meier failure analysis curves demonstrate the cumulative incidence of instrumentation revision surgery following the identification of pelvic screw loosening compared to cases without evidence of loosening. The number of remaining cases at risk are listed below each timepoint to account for censoring of cases without further follow-up

Kaplan–Meier curves for instrumentation revision surgery were generated for cases with and without evidence of pelvic screw loosening **(**Fig. 4**)**. Pelvic screw loosening was associated with a significantly worse failure function curve (*p* = 0.027, Log-rank test).

Patients with evidence of pelvic screw loosening, irrespective of need for eventual revision, did not report significantly worse PROMIS Pain Interference (mean difference 1.1, 95% CI − 3.0 to 5.3) or Physical Function (mean difference − 1.2, 95% CI − 5.2 to 2.9) T-Scores compared to individuals without loosening.

## Discussion

In this retrospective cohort of patients who underwent long-segment fusion for correction of ASD, we assessed the incidence and clinical outcomes of radiographic loosening of spinopelvic fixation. In contrast to acute instrumentation failure, which can be quite dramatic, delayed pelvic screw loosening can develop insidiously with unclear clinical significance. On review of serial follow-up standing radiographs, evidence of pelvic screw loosening was eventually appreciated in over 15% of the patients and often developed years following surgery. Notably, this finding is consistent with high rates of distal construct loosening reported in similar ASD cohorts [8–10, 17–20]. Identification of pelvic screw loosening was associated with significantly increased odds of instrumentation revision surgery, including specifically revision of spinopelvic fixation. Despite the increased odds of delayed instrumentation failure, among the subset of cases with available PROMIS T-scores, pelvic loosening was not associated with significantly worse patient-reported pain or physical function. Notably, approximately half of the cases with evidence of pelvic screw loosening did not ultimately require revision surgery. In this way, spinopelvic loosening, alone, may not drive significant clinical impairment, but rather identify patients at risk for progression to clinically relevant instrumentation failure requiring revision surgery. As such, patients with identified loosening may warrant increased attention and more frequent surveillance in outpatient follow-up.

Various patient and surgical factors have been evaluated in association with rates of spinopelvic fixation failure. Patient-related factors can be associated with increased risk of complications following deformity corrections. In this way, patient selection and pre-operative optimization afford an opportunity to minimize the risk of instrumentation failure. Notably, osteoporosis was associated with higher rates of spinopelvic fixation loosening in our cohort, as may be expected and has been reported elsewhere [20]. Osteoporosis is known to be associated with significantly higher rates of instrumentation failure [21] and as such should be considered carefully in the adult spinal deformity population. While screening and practice patterns were not uniform across the multiple surgeons comprising our cohort, the standard practice of the senior author is to pre-treat osteoporotic patients with osteoanabolic agents. Specifically, teriparatide has been suggested to reduce the likelihood of instrumentation failure, including pseudoarthrosis, in adult deformity patients with osteoporosis [22]. Smoking is also clearly associated with increased rates of fusion failure [23]. As may be expected, within our cohort patients requiring revision surgery were more likely to be smokers. Addressing this modifiable risk factor pre-operatively provides an important opportunity to mitigate the risk of instrumentation failure.

While L5-S1 interbody fusion has been suggested to reduce the incidence of spinopelvic screw loosening [9], other studies such as ours have failed to observe a significant difference [24]. This observation in our cohort may be explained by the fact that although interbody cage placement may support the development of a solid segmental fusion across the L5-S1 disc space, pelvic screw loosening can still develop due to lack of fusion across the sacroiliac joint. As such, pelvic screw loosening should not be conflated with L5-S1 pseudoarthrosis. Similarly, conflicting reports exist concerning the impact of multi-rod constructs with some describing increased rates of loosening of pelvic fixation [24] and others found multi-rod constructs to be protective [25]. Supplementary pelvic screws have been reported to be associated with lower rates of loosening [26, 27], although we did not appreciate a significant difference. In our cohort, pelvic fixation was performed utilizing traditional iliac screws, S2AI screws or a combination of iliac and S2AI screws. Interestingly, while rates of pelvic screw loosening did not significantly differ by method of fixation, rates of instrumentation revision surgery were twice as high in cases with only traditional iliac screws compared to those utilizing S2AI screws. Multiple studies have specifically assessed differences in outcomes between iliac and S2AI screws, with mixed results. Meta-analyses have reported significantly higher odds of screw loosening and revision surgeries with iliac screws compared to S2AI [28, 29]. Meanwhile, other cohorts have reported increased odds of loosening with S2AI screws, but without a significant difference in rates of revision surgery [10]. The intriguing observation of a reduced surgical revision rate in patients with S2AI screws, despite a comparable rate of radiographic loosening to traditional iliac screws, warrants consideration. While our study was not designed to assess this particular question, one may speculate on factors driving the observed difference. Painful instrumentation is a frequent indication for surgical revision. The more medial and deeper entry point of S2AI screws may allow for greater soft tissue coverage than traditional iliac screws, reducing the likelihood of symptomatic prominent instrumentation. Additionally, S2AI screws traverse multiple cortical surfaces across the sacroiliac joint, which may mitigate observed radiographic lucency or promote a differential mode of failure than traditional iliac screws. Further understanding of the patient and surgical factors associated with instrumentation failure, including the selection of pelvic fixation methodology, will be important in guiding pre-operative patient selection and developing surgical approaches to optimize patient outcomes.

Limitations include the retrospective single-institution nature of our study. Cases were sourced from routine clinical practice and outcome measures were not prospectively collected in a strictly scheduled fashion. As a result, PROMIS scores were available for only a subset of cases, consistent with known significant challenges associated with successful collection of patient-reported outcomes in routine clinical practice [30, 31]. As such, PROMIS measures were considered as a secondary outcome in our study, with the possibility that the subset of patients with available data might not perfectly reflect the wider cohort.

Although included cases required a minimum of two-year follow-up, the duration of follow-up was variable. Our data support the understanding that instrumentation failure and revision surgery can occur years following deformity correction surgery. To account for this natural history, we established a minimum two-year follow up for study inclusion, with a resulting median follow-up greater than four years. Patients requiring revision surgery were followed for a longer duration, as one might expect given the more complicated clinical course, and consistent with similar previously reported cohorts [32]. Still the possibility remains that increased duration of follow-up is associated with increased opportunity for identification of spinopelvic fixation failure leading to revision.

Future efforts should focus on approaches to minimize rates of spinopelvic screw loosening and associated instrumentation revision surgery. Identification of non-modifiable patient-specific risk factors will assist in patient selection, whereas identification of modifiable risk factors may allow for pre-operative intervention and optimization [33]. The impact of intra-operative real-time assessment of adequacy of deformity correction should also be considered. Achieving optimal, age-appropriate correction of spinal deformity has been associated with improved post-operative outcomes and reduced rates of instrumentation failure [18, 19, 25, 34]. The impact of adequacy and magnitude of deformity correction on the development of screw loosening warrants further consideration.

## Conclusions

Long-segment fusion for the correction of ASD is associated with a high rate of instrumentation failure, often requiring surgical revision. Loosening of spinopelvic fixation may be identified on follow-up radiographs years following surgery. While such loosening may reflect an incidental finding, loosening of pelvic screws was associated with significantly increased odds of eventual instrumentation revision surgery. As such, identification of spinopelvic fixation loosening may serve as a harbinger of potential impending spinal instrumentation failure, warranting increased attention and continued close follow-up. Spinopelvic fixation techniques, such as traditional iliac screws vs S2AI screws, may influence the likelihood of eventual instrumentation failure and need for revision surgery.

## Data Availability

The data supporting the findings of this study are available from the corresponding author upon reasonable request.
